# Gut microbiota and its association with gastrointestinal symptoms and pharmacological treatments in a sibling-matched cohort with autism spectrum disorder

**DOI:** 10.3389/frmbi.2026.1777385

**Published:** 2026-03-24

**Authors:** Florencia Peñalba, Andreina Guisande, Lucía Lamberti, Camila Rusiñol, Maite Irastorza, Florencia Konik, Claudio Iglesias, Paula Mendive, Gabriela Garrido, Andrés Parada, Nadia Riera

**Affiliations:** 1 Microbial Genomics Laboratory, Institut Pasteur de Montevideo, Montevideo, Uruguay; 2 Department of Pediatric Gastroenterology, Hepatology and Nutrition, Pereira Rossell Hospital Center, Montevideo, Uruguay; 3 Academic Unit of Pediatric Psychiatry, School of Medicine, University of the Republic, Montevideo, Uruguay; 4 Unidad Académica (U.A.) Área de Investigación, Escuela de Nutrición, Universidad de la República, Montevideo, Uruguay; 5 Center for Innovation in Epidemiological Surveillance, Institut Pasteur Montevideo, Montevideo, Uruguay

**Keywords:** 16S rRNA, autism spectrum disorder (ASD), gastrointestinal symptoms (GI), microbiome, oxford nanopore amplicon sequencing

## Abstract

Autism Spectrum Disorder (ASD) is a complex neurodevelopmental disorder marked by difficulties in communication, social interaction, and restricted, repetitive behaviors. The gut microbiota has emerged as a key factor in the gut-brain axis relevant to ASD. We conducted a cross-sectional study comparing the gut bacterial composition of children with ASD (n=29) and their neurotypical siblings (NT, n=29). To minimize environmental and lifestyle confounders, all pairs were 4 to 10 years old and cohabiting in the same household in Uruguay. We used full-length 16S rRNA gene (V1–V9) sequencing with the latest R10.4.1 Oxford Nanopore Technologies chemistry, enabling high-resolution microbial characterization. While overall β-diversity did not differ significantly between the ASD and NT groups, we identified specific taxonomic shifts. The ASD group was enriched in taxa like *Sellimonas*, while the NT group showed enrichment of genera like *Faecalibacterium* and *Coprococcus*. Furthermore, we found GI symptoms to be significantly more prevalent in the ASD group and some bacterial genera associated with GI symptomatology. In addition, we explored the association of pharmacological treatments. Antipsychotic use was associated with reduced *Akkermansia* abundance, whereas melatonin and methylphenidate use were associated with the enrichment of *Negativibacillus*. This study provides novel insights into the gut microbiome of Uruguayan children with ASD, delineating the influence of GI symptoms and pharmacological load on microbial diversity and composition.

## 1 Introduction

Autism Spectrum Disorder (ASD) encompasses a highly heterogeneous group of neurodevelopmental conditions characterized by difficulties in communication and social interaction. Additional core features include restricted interests and repetitive behaviors. The incidence of ASD has been rising globally in recent years, with a median prevalence currently estimated at approximately 0.72% (Talantseva et al., 2023). This increase can be attributed not only to more advanced diagnostic methods, but also to the growing prevalence of associated risk factors (Arango et al., 2021; Solmi et al., 2022). Furthermore, children with ASD are characterized by a high co-occurrence of several mental and medical conditions, including attention deficit hyperactivity disorder (ADHD), anxiety, depression, phobias, intellectual disability, speech and language impairments, sleep disturbances, restrictive or avoidant food intake, and gastrointestinal (GI) symptoms (Leyfer et al., 2006; Simonoff et al., 2008; Doshi-Velez et al., 2014). In particular, the co-occurrence of ASD and GI problems (including constipation, diarrhea, and abdominal pain) is widely recognized (Wasilewska and Klukowski, 2015), although the exact reported prevalence varies between studies (Molloy and Manning-Courtney, 2003; Valicenti-McDermott et al., 2006; Ming et al., 2008). Despite this high frequency, the underlying mechanisms linking GI disturbances and ASD remain poorly understood.

The gut microbiome, defined as the complex community of microorganisms, including bacteria, archaea, viruses, and fungi, that inhabit the gastrointestinal tract has attracted substantial research interest in the context of ASD. Microbial-derived metabolites (substances produced by bacteria), such as short-chain fatty acids (SCFAs) and neurotransmitters, facilitate bidirectional communication with the central nervous system in a bidirectional manner called the microbiota-gut-brain axis (Jennis et al., 2018). Moreover, the microbiome is a highly complex ecosystem influenced by both genetic and environmental factors, such as diet, culture, lifestyle and geographic region (Bajinka et al., 2020; Rackerby et al., 2023; Fackelmann et al., 2025; Zeng et al., 2025). It has been consistently reported in numerous studies that individuals with ASD exhibit a distinctive gut microbiome compared to neurotypicals (NT). Notably, these variations are observed in the bacterial community structure and the resulting metabolomic production. However, there is significant inconsistency among the results published (Iglesias-Vázquez et al., 2020), often finding different taxa correlated with ASD or even presenting opposite results in microbial signatures.

The variance in the microbiota composition of children with ASD could be attributed to the heterogeneity of this disorder. ASD is a complex condition encompassing a wide array of behavioral characteristics and severity levels that may translate into diverse microbial profiles. Furthermore, inconsistencies are compounded by differences in research methodology and external factors, including variations in sample collection protocols, sample size, as well as crucial environmental variables such as diet, culture and the location (Mitchell et al., 2025). This clinical complexity and methodological inconsistency makes it extremely difficult to establish a single, unique result that can be established for the condition. Additionally, research into the role of microbes in human health has largely focused on North American and European countries (Abdill et al., 2022), leaving Latin America notoriously underrepresented.

Although there is still no specific pharmacological treatment for the core symptoms of ASD, co-occurring conditions are often managed with pharmacological treatments that can be categorized into atypical antipsychotics, stimulants, antidepressants, among others. Several studies have suggested that these pharmacological treatments may influence the gut microbiota composition, highlighting the importance of considering medication use when interpreting microbiome profiles in this population (Bahr et al., 2015; Ren et al., 2017; Gao et al., 2020; Kim et al., 2020).

In this work, we conducted a cross-sectional study of children with ASD (*n* = 29) and their neurotypical siblings (*n* = 29), living in the same household and within the same age range (4–10 years). We profiled the gut bacterial composition using full-length 16S rRNA gene (V1–V9) sequencing with the latest R10.4.1 Oxford Nanopore Technologies chemistry, which enables high-resolution microbial characterization and, in many cases, strain-level discrimination (Wagner et al., 2016; Johnson et al., 2019; Zhang et al., 2023). Studying cohabiting sibling pairs provides a robust framework for examining gut microbiome differences while minimizing environmental and lifestyle confounders, particularly in an underrepresented Latin American population. Therefore, this study aimed to characterize the gut microbiota of children with ASD compared to their neurotypical siblings, evaluating differences in alpha-diversity, beta-diversity, and taxonomic composition at the family and genus levels. Furthermore, we sought to evaluate associations between specific bacterial taxa and both gastrointestinal symptoms and pharmacological treatments, with the ultimate goal of identifying potential microbial biomarkers associated with ASD.

## 2 Materials and methods

### 2.1 Study design and patient selection

The study cohort consisted of two groups totaling 58 children, each between 4 and 10 years of age. One group included 29 children diagnosed with autism spectrum disorder (ASD), while the second group comprised their neurotypical siblings, serving as matched home cohabitant controls. Exclusion criteria included a diagnosis of attention-deficit/hyperactivity disorder (ADHD), diabetes mellitus, genetic diseases, inborn errors of metabolism, inflammatory bowel disease, celiac disease, and motor disability. The diagnosis of ASD was confirmed by qualified professionals following the *Diagnostic and Statistical Manual of Mental Disorders, Fifth Edition (DSM-5)* guidelines (Wiggins et al., 2019).

### 2.2 Gastrointestinal symptoms

Additionally, each family completed a gastrointestinal symptom questionnaire to assess the presence and severity of digestive issues in the children conducted by the Unidad de Psiquiatría of the Hospital Pereira Rosell, Montevideo, Uruguay. The questionnaire was collected by a Child Gastroenterologist and completed by the children’s caregivers or guardians to gather detailed information on the presence, frequency, and severity of symptoms and has been previously validated (Chaidez et al., 2014) GI symptoms included abdominal pain, abdominal distension, meteorism, constipation, food sensitivity, swallowing disorder, defecatory pain, vomiting and diarrhea, the questionnaire was adapted to Spanish. Current pharmacological treatments were documented during the interview, with a particular focus on melatonin, antipsychotics, and stimulants (e.g., methylphenidate), among others.

### 2.3 DNA extraction

Fecal samples were collected from each child in sterile stool collection containers and immediately stored at −80 °C until DNA extraction. Genomic DNA was extracted from all 60 samples using the QIAamp PowerFecal Pro DNA Kit (Qiagen, CA, USA), following the manufacturer’s instructions. DNA concentration was measured with a fluorimeter Qubit 2.0 (Invitrogen) using the Qubit High Sensitivity DNA Assay Kit (Invitrogen, CA, USA), and DNA integrity was evaluated by agarose gel electrophoresis 0.8%. Extracted DNA was stored at −20 °C until further processing for sequencing.

### 2.4 PCR amplification of long reads 16S rRNA and nanopore sequencing

Approximately 10 ng of extracted genomic DNA per sample was used for PCR amplification of the full-length 16S rRNA gene, targeting the V1–V9 hypervariable regions. Amplification was performed using custom-modified primers adapted for Oxford Nanopore Sequencing, 27F (5´TTTCTGTTGGTGCTGATATTGCAGRGTTYGATYMTGGCTCAG3´) and 1492R (5´ACTTGCCTGTCGCTCTATCTTCRGYTACCTTGTTACGACTT3´). Long-read libraries were prepared following the Oxford Nanopore Technologies (ONT) protocol using the Ligation Sequencing Kit (SQK-LSK114) in combination with the Native Barcoding Expansion 96 (EXP-NBD196). Sequencing was carried out on a Gridion platform. Basecalling was carried out during the sequencing run using high-accuracy model v3.3, 450 bps. This basecalling model coupled with the R10.4 latest chemistry of ONT used in this study typically results in Phread Scores of ~Q20. Raw signal data (POD5 files) were processed to produce high-quality reads in FASTQ format with a mean quality score > 7.

### 2.5 Bioinformatic analysis

Raw long-read sequences were processed and qualified using Porefile (microgenlab, 2025) with the SILVA 138.1 database. The resulting read count table, taxonomic classification, and sample metadata were integrated into a *phyloseq* object for downstream analysis using the *phyloseq* package (1.52.0) (McMurdie and Holmes, 2013) in Rstudio (v 4.5.1) (Posit Team, 2025) and R (v 4.4.3) (R Core Team, 2016). Taxa abundances were subsequently aggregated at the genus level. Alpha diversity was calculated using the Shannon index, implemented through the estimate_richness function in *phyloseq*. Beta diversity was assessed based on Bray–Curtis dissimilarity, and principal coordinate analysis (PCoA) was performed to visualize differences in microbial community composition among samples. All code used in this study is available at Github repository at https://github.com/fpenalba09.

### 2.6 Statistical analysis

Statistical differences between groups in alpha diversity were tested using the Wilcoxon rank-sum test implemented in the *rstatix* (Kassambara, 2025) package. A permutational multivariate analysis of variance (PERMANOVA) was conducted using the *adonis2* function from the vegan (Oksanen et al., 2001) package in R to evaluate differences in microbiota composition based on Bray–Curtis similarity. Additionally, Linear Discriminant Analysis Effect Size (LEfSe) (Segata et al., 2011) was applied to compare different groups and identify significantly enriched taxa, considering an absolute LDA score ≥ 3 as the threshold for differential abundance. All statistical analyses were performed using R software. Wilcoxon rank-sum test was used to evaluate differences in gastrointestinal symptoms among groups.

### 2.7 Network analysis

A co-occurrence network analysis was constructed to characterize the relationships among bacterial genera. This network included all genera shared between the cohorts, as well as those uniquely present in either the ASD or NT group. The SparCC (Sparse Correlations for Compositional data) algorithm, implemented in the fASTsPAR package (Watts et al., 2019), was used to calculate correlations based on the genus-level taxonomic data. To determine statistically significant correlations, a two-sided p-value was calculated, and a strict cutoff of p<0.01 was applied. The resulting co-occurrence network was visualized using the *ggraph* package in R (Si et al., 2022).

### 2.8 Microbiota-symptom correlation and clustering

To analyze the relationship between gastrointestinal (GI) symptoms and gut microbiota, we first compared the prevalence of GI symptoms between the ASD and NT cohorts using the Wilcoxon rank-sum test, and visualized these differences with a circos plot function package circlize (Gu, 2025). We then determined the inter-correlation between the incidences of all GI symptoms. Spearman correlation metric was used to assess the relationship between bacterial genera and all symptoms because of its non-parametric and rank-based nature. We employed the corrplot (Taiyun, 2025) function in R which allowed for simultaneous hierarchical clustering of both bacterial genera and symptoms. We applied the false discovery rate (FDR) Benjamini-Hochberg correction to account for multiple testing false positive results (with the p.adjust function from stats). The dendrogram was visually inspected, and the cut height was empirically determined to partition the tree into three distinct branches (Modules 1, 2, and 3). We then defined three distinct modules (clusters) of co-occurring GI symptoms: Module 1 (Food sensitivity): Module 2 (Meteorism, blood in stool, constipation and defecatory pain), Module 3 (Abdominal pain, vomiting, abdominal distension and swallowing disorder). To enable differential abundance testing, the 58 participants were categorized for each module into four clinical groups based on diagnosis and the presence (≥1 symptom) or absence of symptoms: ASD+ (ASD with symptoms), ASD- (ASD without symptoms), NT+ (neurotypical with symptoms), and NT- (neurotypical without symptoms). For Module 1, the group sizes were nASD+=8, nASD−=21, nNT+=1, and nNT−=28. For Module 2, the distribution was nASD+=21, nASD−=8, nNT+=15, and nNT−=14. Finally, for Module 3, the counts were nASD+=20, nASD−=9, nNT+=7, and nNT−=22. These four-group classifications, specific to each module, served as the basis for LEfSe analysis to identify microbial taxa significantly associated with the presence or absence of each respective symptom cluster.

### 2.9 Pharmacological treatments and classification

During the gastrointestinal questionnaire, families were also asked to report about the pharmacological treatments received. Based on this data, we classified all drug treatments into three main categories: sleep aids (melatonin), Central Nervous System Stimulants (methylphenidate), and antipsychotics (risperidone, aripiprazole). Other medications were also documented but the frequency was rare and were not considered in statistical analysis.

## 3 Results

### 3.1 Participant characteristics

The study cohort comprised a total of 58 participants, including 29 children diagnosed with autism spectrum disorder (ASD) and their neurotypical (NT) siblings without a diagnosis. All participants were residents of Uruguay. The ASD group included 29 children aged between 4 and 10 years, of whom 24 were male and 5 were female.The NT group consisted of 29 siblings within the same age range, including males 13 and 16 females.

### 3.2 Differences in the gut microbiota of ASD and their NT sibling

**Figure 1:**
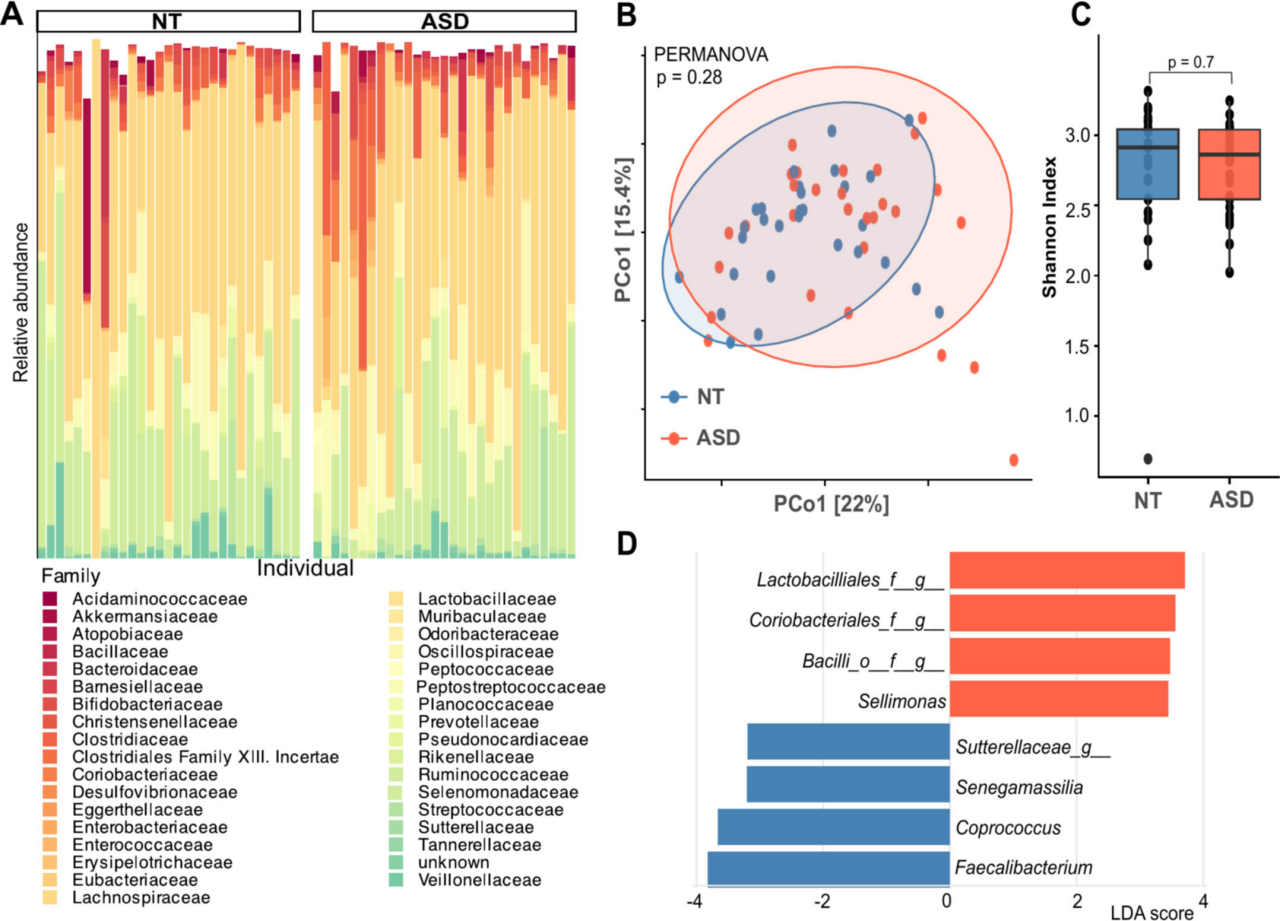
**(A)** Relative abundance at family level for those samples in Autism spectrum disorder (ASD) and Neurotypical (NT) cohorts. **(B)** Beta Diversity estimated with Bray-Curtis dissimilarity distance and visualized as a PCoA (Principal Coordinates Analysis) **(C)** Histogram with Alpha diversity (Shannon Diversity Index) estimates for both cohorts **(D)** Linear Discriminant Analysis Effect Size (LEfSe) results. Linear Discriminant Analysis (LDA) score shows enriched taxa in each group, bars are colored according to these groups.

Sequencing resulted in an average read count of 118,500 reads per sample with a minimum read count of 8,973. We assessed sequencing depth and the recovered microbial ecology by plotting rarefaction curves of each sample, based on the observed plateau of each plot we decided to include all sample results in the comparative analysis. After processing the FAST5 files, Porefile retrieved 230 different bacterial taxa. We determined that the overall bacterial community structure, in both cohorts, encompassed 48 different bacterial families. Crucially, the comparison of taxonomic relative abundance at the Family level was highly resolved, as we could determine the taxonomic identity for more than 97% of all sequencing reads in every sample. This detailed resolution allowed us to identify several significant groups with a differential distribution between the two groups, highlighting key compositional differences in the microbial communities (**Figure 1A**). The families Sutterellaceae and Veillonellaceae exhibited a higher relative abundance in the NT group. In contrast, the family Clostridiaceae was observed to be more abundant in the ASD group. To assess the similarity of the microbial communities between the NT and ASD cohorts, beta diversity was calculated using the Bray-Curtis distance and visualized via Principal Coordinates Analysis (PCoA). We observed no statistical difference between the microbial communities of the two compared groups (PERMANOVA, p=0.28) (**Figure 1B**). Alpha diversity was compared between the two groups at the genus level. Statistical analysis using the Wilcoxon rank-sum test revealed no significant differences in α-diversity metrics between the Neurotypical and Autism Spectrum Disorder groups (**Figure 1C**). Next, we performed a LEfSe (Linear Discriminant Analysis Effect Size). The LDA scores highlighted several enriched taxa in each group (**Figure 1D**; **Supplementary Table 2**). The ASD group of children was characterized by a higher relative abundance of several taxa, including *Bacilli* at class level, Lactobacillales and Coriobacteriales at order levels. Our results show *Sellimonas* as a genus with significantly higher abundance in ASD. On the other hand, the NT group was distinguished by the enrichment of the genera *Faecalibacterium*, *Coprococcus*, *and Senegalimassilia*.

### 3.3 Autistic children and their neurotypical siblings differ in the bacterial community structure but share a common bacterial community core

**Figure 2:**
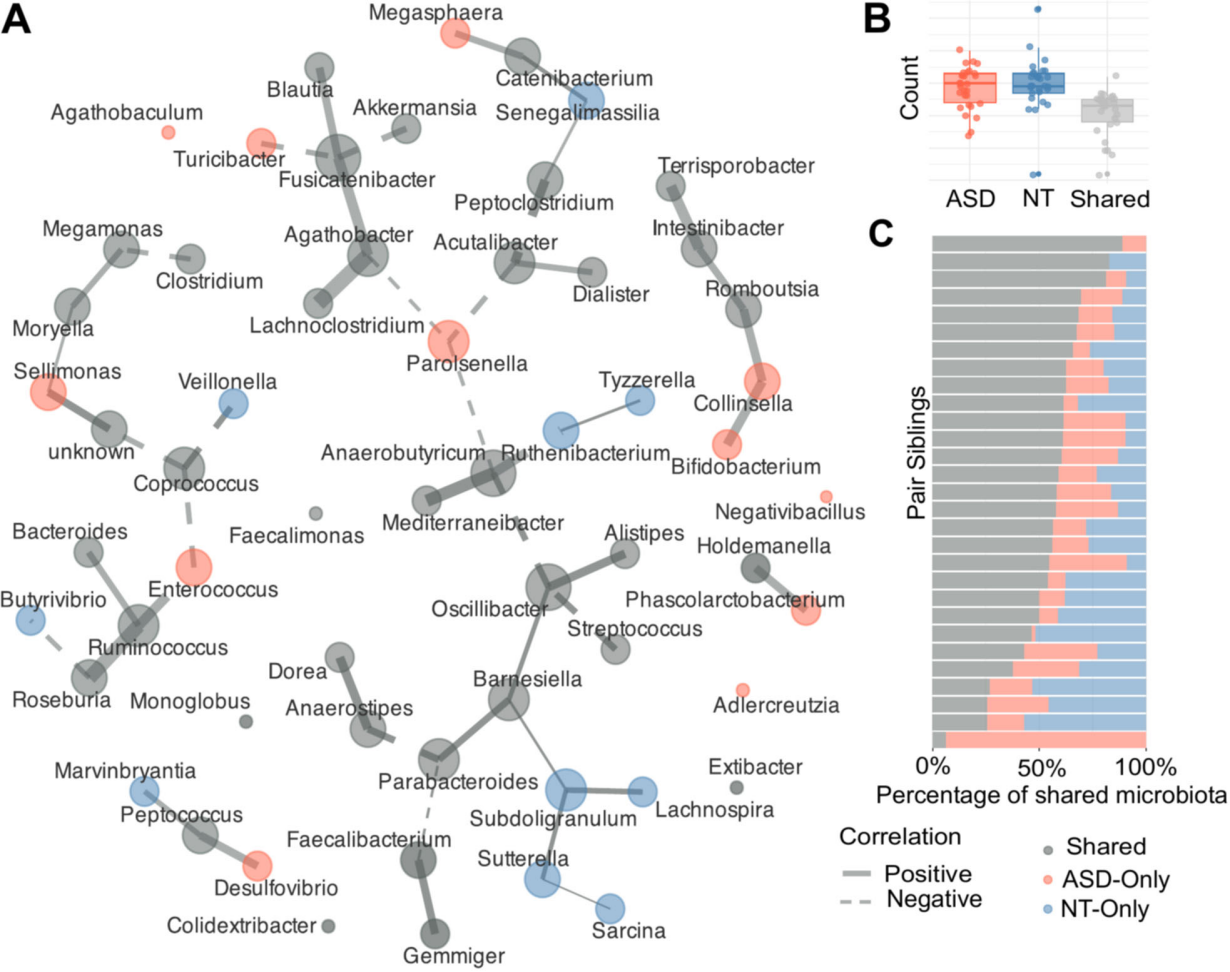
**(A)** Co-occurrence network showing interactions within gut microbiota. Interactions are shown between bacterial genera combining the Autism spectrum disorder (ASD) and Neurotypical (NT) cohorts. Nodes are colored by cohort specificity (green: shared; red: ASD-only; Blue: NT-only) and sized by degree centrality. Edges indicate correlations, with line width representing interaction strength (“r”) and color indicating direction (Solid: positive; Dashed: negative). **(B)** Box-plot representing the count of bacterial genera classified into three groups: Shared (Gray), ASD-Only (Red), and NT-Only (Blue). Each point represents an individual sample. The box displays the interquartile range (IQR), and the line indicates the median proportion for each category. **(C)** Stacked bar chart detailing the compositional breakdown of the gut microbiota for each sibling pair (Y-axis). Each bar is segmented by the percentage of genera belonging to the three categories: Shared (Gray), ASD-Only (Red), and NT-Only (Blue). This panel visually confirms the variability and the high proportion of shared genera across the individual pairs.

To evaluate differences in microbial community interactions between the ASD and NT groups, a co-occurrence network was constructed at the genus level. In this network analysis, we differentiated microbial genera based on whether their significant interactions were shared between the groups (green nodes) or were exclusive to either the ASD (red nodes) or NT (blue nodes) (**Figure 2A**). The analysis demonstrated both structural differences in the microbial interaction networks and a shared core of bacterial genera interactions. This common skeleton includes key genera such as *Faecalibacterium*, *Oscillibacter*, *Akkermansia*, *Roseburia*, *Ruminococcus*, *Mediterraneibacter*, and *Dorea* among others. Interestingly, some of these genera also correspond to those identified as significantly enriched in NT such as *Faecalibacterium*, *Coprococus*, *Senegalimassilia* (**Figure 1D**) further pointing at differences in bacterial abundance rather than bacterial composition among the two groups. This finding suggests that the gut microbiomes of ASD and NT children partly differ in their interaction between bacterial groups. Bacteria only present in the ASD group are members of the *Enterococcus*, *Sellimonas*, *Parabacteroides* and *Negativibacillus* families. While *Butyrivibrio*, *Sutterella*, *Senegalimassilia*, *Lachnospira* and *Alistipes* were identified only in the NT group. A pronounced observation was the tendency for negative interactions (dashed red lines) to be driven by ASD-exclusive genera. Specifically, the ASD-exclusive genera *Enterococcus* and *Sellimonas* exhibited negative correlations with *Ruminococcus*, *Coprococcus*, and *Moryella*. Similarly, *Turicibacter* (ASD-exclusive) showed a strong competitive link with *Blautia*, and *Parasolnella* (ASD-exclusive) was negatively correlated with multiple genera including *Anaerobactyrium*, *Acutalibacter*, and *Agathobacter*. In our analysis, positive interactions were predominantly clustered around those genera that were shared between the two groups or were exclusive to the NT group. Upon examining the distribution of genera across groups, we quantified the number of genera that were exclusive to each group as well as those that were shared between ASD and NT. The boxplot indicates that ASD and NT children harbor a comparable but distinct number of unique genera, with both groups showing greater counts than the shared subset. This pattern suggests possible differences in the gut microbiome composition of ASD and NT children (**Figure 2B**). To assess whether this pattern was also present within sibling pairs, we determined the proportion of genera that were shared within each pair and those that were present exclusively in one sibling. A substantial proportion of genera was shared within each pair but also some that were exclusively from the groups. It is important to note that this comparison was performed at the genus level, and therefore we cannot determine whether siblings truly harbor the same bacterial strains. Further strain-level analyses will be needed to clarify the extent of microbial sharing within sibling pairs (**Figure 2C**).

### 3.4 Association between gastrointestinal symptoms and differential abundance in microbial taxa

**Figure 3:**
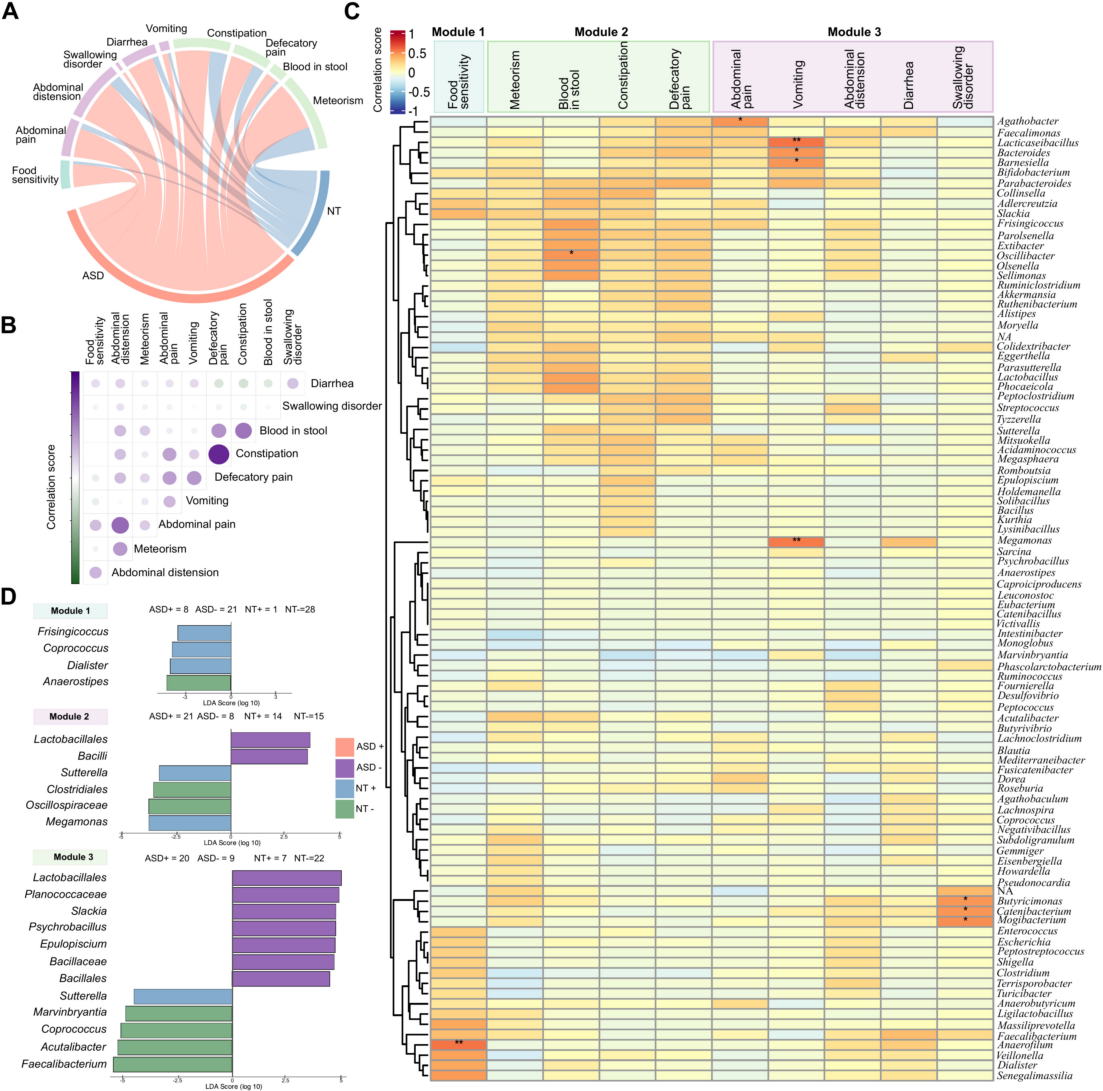
**(A)** Circos plot illustrating the relationships and co-occurrence patterns between different gastrointestinal symptoms. **(B)** Correlation matrix quantifying the pairwise relationships between symptoms. The color scale (Green to Purple) and circle size indicate the direction and strength of the correlation, with purple representing strong positive associations (co-occurrence) and green representing negative associations. **(C)** Heatmap showing the correlation between GI symptoms. Those cells with asterisk denote significant correlations (*p values < 0.05; **p values < 0.01) **(D)** LEfSe (Linear Discriminant Analysis Effect Size) analysis among the modules found in the (see also **Figure 4** below). LDA (Linear Discriminant Analysis) score shows enriched taxa within each module.

When assessing the prevalence of GI symptoms, several symptoms were found to be significantly more predominant in the Autism Spectrum Disorder (ASD) group compared to the Neurotypical (NT) group. Specifically, abdominal distension, abdominal pain, meteorism, diarrhea, and food sensitivity were reported at significantly higher rates in children with ASD (**Supplementary Figure 1**). The frequency of other symptoms, including defecation pain, vomiting, constipation, and swallowing disorder were also more prevalent in the ASD group but showed no statistically significant difference (Wilcoxon test, p>0.05)(**Supplementary Figure 2**). The overall prevalence of gastrointestinal symptoms in the ASD group shows a higher frequency compared to the NT group (**Figure 3A**). We then explored how these symptoms correlated with each by estimating the Spearman rank correlation coefficient (**Figure 3B**).

The analysis of symptom correlations revealed several distinct clusters of related co-occurring gastrointestinal variables. For instance, defecatory pain showed a strong association with the presence of blood in stool and constipation. Furthermore, abdominal distension was broadly correlated with abdominal pain, diarrhea and swallowing disorder. Separately, the acute symptoms of diarrhea and vomiting were found to be linked, as these are common during infectious or inflammatory events. Finally, meteorism also exhibited a significant correlation with abdominal distension. The Spearman correlation analysis revealed distinct microbial signatures associated with specific gastrointestinal phenotypes, effectively clustering bacterial genus abundances with clinical symptom profiles. A highly significant positive correlation was observed between food sensitivity and the genus Anaerofilum. Additionally, significant positive associations were identified with *Senegamassilia*, *Dialister*, *Veillonella*, and *Faecalibacterium*. Symptoms related to intestinal transit and local irritation (Module 2) demonstrated a consistent cluster of positive correlations. Notably, the genera *Oscillibacter*, *Olsonella*, and *Sellimonas* exhibited robust positive associations within this cluster, remaining largely uncorrelated with other gastrointestinal phenotypes. *Oscillibacter*, in particular, showed a statistically significant link with the presence of blood in stool. Vomiting showed highly significant positive correlations with *Lacticaseibacillus* and *Megamonas*, as well as significant associations with *Faecalimonas*, *Bacteroides*, and *Barnesiella*. Furthermore, abdominal pain was specifically associated with *Agathobacter*, while swallowing disorders exhibited significant positive correlations with *Butyricimonas*, *Catenibacterium*, and *Mogibacterium*. Conversely, the analysis identified a group of taxa that were negatively correlated with several gastrointestinal symptoms. The genera *Coprococcus*, *Dorea*, and *Roseburia*, often associated with butyrate production and gut health, displayed inverse relationships with the clinical profiles (**Figure 3C**).

Conversely, the genera enriched in the NT group (*Faecalibacterium* and *Coprococcus*) were negatively correlated with all the symptoms (**Figure 1D**). Based on the hierarchical clustering applied to the microbiota-symptom correlation matrix we classified the gastrointestinal (GI) symptom in three modules (see Materials and Methods). Focusing on Module 1, the genera *Frisingicoccus*, *Coprococcus*, and *Dialister* were found to be significantly associated with the NT+ group, while *Anaerostipes* was associated with the NT− group. In the analysis of Module 2, the Lactobacillales order and the Bacilli class were significantly associated with the ASD− condition. The genus *Sutterella* and *Megamonas* were associated with the NT+ group. Conversely, the NT− group (neurotypical children without Module 2 symptoms) showed significant associations with Clostridiales and Oscillospiraceae. For Module 3, several taxa were associated with the ASD− group, including *Lactobacillales*, Planococcaceae, *Slackia*, *Psychrobacillus*, *Epulopiscium*, Bacillaceae, and Bacillales. The genus *Sutterella* was associated with the NT+ group. In contrast, the NT− group showed associations with multiple genera, including *Marvinbryantia*, *Coprococcus*, *Acutalibacter*, and *Faecalibacterium* (**Figure 3D**).

### 3.5 Gut microbioma composition association with the use of antipsychotics, melatonin and central nervous system stimulant methylphenidate

**Figure 4:**
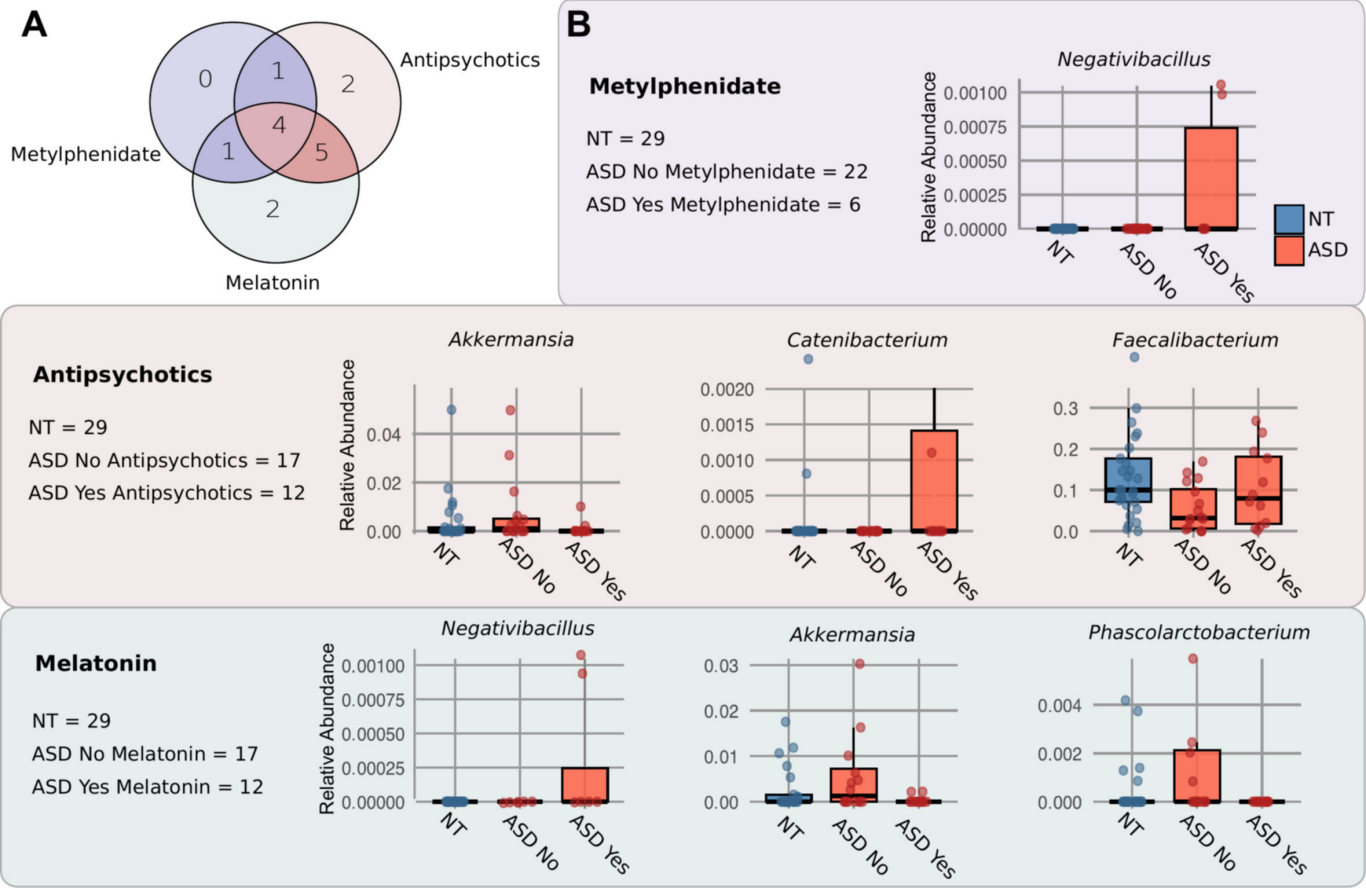
**(A)** The concurrent use of antipsychotics, melatonin and methylphenidate is shown as a Venn diagram. Numbers indicate the counts of patients in each intersection. **(B)** Following a LEfSe (Linear Discriminant Analysis Effect Size) analysis, the histograms show significant differential normalized abundance of taxa associated with different consumption of medication. **(B)** The concurrent use of antipsychotics, melatonin and methylphenidate is shown as a Venn diagram. Numbers indicate the counts of patients in each intersection.

In the evaluated cohort of 29 children with ASD, 17 were undertaking pharmacological treatments. Twelve children (approximately 41.4%) were taking antipsychotic medication, and the same number were using melatonin, while six children (20.7%) were being treated with methylphenidate. Other medications, such as antiallergic and anti-asthma drugs, were also used by four children. The data highlights a strong trend toward polypharmacy: 13 children (76.5% of the treated subgroup) were undertaking two or more concurrent treatments, with the most frequent specific combination being Antipsychotics and Melatonin (taken by four children exclusively) (**Figure 4A**; **Supplementary Table 1**). Neurotypical siblings in the control group did not receive pharmacological treatments in these categories and were thus used as a control group.

To determine the potential impact of the diverse and often combined treatments on the gut microbiome, a LEfSe analysis was performed to identify bacterial taxa that differed significantly between children who took or not each specific medication (**Supplementary Table 2**). Firstly, children undertaking melatonin were evaluated. Our results showed that *Negativibacillus* was enriched in ASD children who consumed melatonin, whereas *Akkermansia* and *Phascolarctobacterium* were enriched in those who did not undertake melatonin. When comparing these genera across groups, *Negativibacillus* showed higher abundance in ASD children consuming melatonin compared with both NT children and ASD children who did not take melatonin. In contrast, *Akkermansia* appeared to be more abundant in NT children than in ASD children treated with melatonin, whereas ASD children who did not undertake melatonin showed the highest abundance of *Akkermansia* among the three groups (**Figure 4B**). A similar pattern was observed for the genus *Phascolarctobacterium*. LEfSe analysis of children undertaking methylphenidate identified a single enriched taxon: *Negativibacillus*, which was more abundant in children consuming this medication. In contrast, both NT children and ASD children who did not undertaking methylphenidate showed no detectable abundance of this genus. Regarding antipsychotic use, significant associations were observed with *Faecalibacterium* and *Catenibacterium*, which were more abundant in children under antipsychotic treatment, whereas *Akkermansia* was enriched in those who did not undertake it. Specifically, *Faecalibacterium* displayed comparable mean abundance in NT children and ASD children treated with antipsychotics, while ASD children who did not undertake this medication exhibited markedly lower levels. In the case of *Akkermansia*, higher abundances were observed in NT children and ASD children without antipsychotic treatment, whereas ASD children undertaking antipsychotics showed reduced levels.

A smaller subset of seven children (25%) reported the use of homeopathy. At the genus level, in the subgroup of children undertaking homeopathy, three genera, *Mogibacterium*, *Colidextribacter*, and *Alistipes*, were found to be more abundant compared with those not undertaking homeopathy (**Supplementary Figure 3**). When comparing the abundance of *Mogibacterium* across groups, we observed that it was higher in ASD children undertaking homeopathic treatment compared with the NT group. *Colidextribacter* tended to be more abundant in NT children than in ASD children who did not undertake homeopathy; however, its abundance increased in ASD children who did. Similarly, *Alistipes* showed greater abundance in the subgroup of ASD children undertaking homeopathic treatment. In contrast, the ASD group not undertaking homeopathy exhibited lower levels of both *Colidextribacter* and *Alistipes* compared with the NT cohort (**Figure 4A**).

## 4 Discussion

Previous studies have examined differences in the gut microbiota between children with ASD and NT (Kang et al., 2017; Wang et al., 2019, 2023; Ye et al., 2021; Zuffa et al., 2023; Chang et al., 2024) However, factors such as diet, age, daily habits, and environmental exposures can shape the gut microbiome, introducing substantial variability (Rothschild et al., 2018). By comparing children with ASD to their neurotypical (NT) siblings—who share a genetic background and a household environment—we aimed to minimize these confounders and provide a clearer understanding of microbiome alterations associated with the disorder. To our knowledge, this is the first study in Latin America to employ a sibling-pair design and utilize third-generation Nanopore sequencing, providing enhanced taxonomic resolution for characterization (Zhang et al., 2023; Kim et al., 2024).

Our results identified significant microbial shifts, specifically a depletion of butyrate-producing bacteria such as *Faecalibacterium* and *Coprococcus* in the ASD group. This aligns with global trends associating these taxa with healthy gut homeostasis (Zhao et al., 2023). Interestingly, a previous national study conducted in a Uruguayan cohort which compared children with autism (*n* = 30) and neurotypical controls (*n* = 28) within a similar age range (3 to 10 years old) reported differences in *Roseburia* genus between ASD and NT overweight groups. The same study reports *Faecalibacterium prausnitzii* being significantly decreased in the ASD with overweight group which is consistent with our results of higher *Faecalibacterium* genus in NT (Mendive Dubourdieu and Guerendiain, 2023). Our study identified *Coprococcus* as a genus enriched in the NT group. In contrast with our findings, a study conducted on mestizo children in Quito, Ecuador reported an enrichment of the genus *Coprococcus* (along with *Bacteroides*, *Akkermansia*, and *Ruminococcus*) in children with ASD compared to their matched controls (Zurita et al., 2020). Of relevance, a recent study reported opposite results (Notting et al., 2023). This pattern contrasts with results from studies conducted in Western populations, which have consistently described a reduced abundance of *Coprococcus* in children with ASD (Kang et al., 2013, 2018; Coretti et al., 2018). Conversely, the enrichment of *Sellimonas* in our ASD cohort aligns with prior research linking this genus not only to autism but also to other gut-brain axis disorders, such as schizophrenia (Nakhal et al., 2024). The variations and inconsistency across microbiome studies, where associations identified in one cohort may appear in the opposite direction in another, highlight the complexity of interpreting ASD-related microbial patterns (Mitchell et al., 2025). This underscores the importance of considering additional contextual factors such as sex, age, diet, and environmental exposures, which may help explain these cohort-specific differences. For this reason, it is essential to interpret and report microbiome findings with caution, acknowledging the influence of these variables on the observed results.

The depletion of butyrate producers suggests a potential functional deficit in our ASD cohort. Butyrate, a short-chain fatty acid (SCFA) derived from dietary fiber fermentation, plays a vital role in maintaining intestinal barrier integrity and reducing neuroinflammation (Zou et al., 2020). In addition, butyrate significantly influences gut microbial activity, exerting both direct and indirect effects through G-protein–coupled receptors and epigenetic mechanisms, particularly via the modulation of histone deacetylase (HDAC) activity. Dysregulation of this mechanism is frequently implicated in neurological conditions, including Autism Spectrum Disorder (Stilling et al., 2014, 2016). These differences highlight group-specific variations in the microbial network structure. Our network analysis which identified potential butyrate producers like *Butyrivibrio* and *Subdoligranulum* as uniquely present in the NT co-occurrence map, suggesting a more robust metabolic network for SCFA production in neurotypical siblings. This genus showed strong co-occurrence associations with *Lachnospira* and *Sutterella*, suggesting a closely linked metabolic or ecological within the NT gut environment. Furthermore, a significant negative correlation was observed for the genus *Coprococcus* with *Enterococcus* only present identified in the ASD cohort network. Furthermore, the enrichment of *Senegalimassilia* in NT children is particularly intriguing, as this genus is involved in the production of S-equol, a modulator of ERβ receptors which are critical for neurodevelopment and neurotransmission. ERβ is abundantly expressed in key brain regions involved in neurodevelopment, including the cerebral cortex, hippocampus, and cerebellum, where it plays essential roles such as the neurotransmission of dopamine and serotonin (Bendis et al., 2024). While speculative, these findings suggest that the NT microbiome may offer protective metabolic benefits that are less prevalent in their ASD siblings.

Consistent with previous literature, children in the ASD group had a higher prevalence of gastrointestinal symptoms (93%) than their NT siblings (58%). We observed a higher prevalence of autistic children in all gastrointestinal symptoms studied including constipation (**Figure 3A**), however, not all symptoms showed significant differences (**Supplementary Figure 2**). We studied the correlation between microbial genera and gastrointestinal (GI) symptoms and distinct microbial patterns were observed. Notably, the absence of abdominal distension, abdominal pain, and food sensitivity in the ASD group was linked to the enrichment of the genus *Akkermansia*. A study by Cruz-Aguilar et al. highlighted the potential role of *Akkermansia muciniphila* in the reduction of abdominal distension and pain in a cohort with irritable bowel syndrome after FMT of healthy donors. Conversely, the presence of these symptoms in ASD children was associated with the genus *Acutalibacter*. Our observation that *Sutterella* was associated with the absence of symptoms in the NT group contrasts with earlier reports linking this genus to GI distress (Williams et al., 2012). This discrepancy may be due to our use of fecal samples rather than intestinal biopsies, highlighting the impact of sampling site on microbiome findings (Williams et al., 2012).

Finally, we addressed the impact of pharmacological load. Consistent with previous reports, we found that children receiving antipsychotics (such as risperidone) exhibited a marked reduction in *Akkermansia* (Bahr et al., 2015). Also, autism frequently co-exists with sleep disorders, and melatonin use is therefore common among the ASD group (41% in our cohort). While some literature suggests melatonin may increase *Akkermansia* abundance (Hagi and Belzer, 2021), our cohort showed the opposite; however, this is likely explained by polypharmacy, as most children taking melatonin were also receiving antipsychotics. Most of the children who are undertaking melatonin are simultaneously receiving antipsychotics which are themselves known to reduce *Akkermansia* abundance (Bahr et al., 2015). The enrichment of *Negativibacillus* in children taking melatonin or methylphenidate is a novel finding that should be further investigated. Central nervous system stimulants such as methylphenidate are frequently used in children with autism as a way to improve attention and focus and have also been previously reported to alter the gut microbiome of children with Attention-deficit/hyperactivity disorder (Boonchooduang et al., 2025). Further studies are needed to understand the function of the observed changes. It is important to note that the evaluation of medication impacts is particularly susceptible to reverse causation; children presenting with more severe ASD phenotypes or higher GI distress may be more frequently prescribed certain treatments. Consequently, it remains undetermined whether the observed microbial differences are a contributing cause of the clinical phenotypes, a downstream consequence of the disorder’s severity, or a direct result of pharmacological intervention. In our cohort we observed that the ASD group without medication (*n* = 14, 48%) had several GI symptoms (**Supplementary Table 2**). Future work is needed to further understand this association.

## 5 Limitations

Despite the methodological strengths of this study, several limitations must be acknowledged. First, the sample size was relatively small (*n =* 58), which limits the statistical power to detect more subtle microbial shifts. When subgroups were analyzed we presented the number of individuals in each comparison to be aware of this limitation, we acknowledge that in some cases the small sample size results in comparisons substantially underpowered. Therefore, these subgroup associations should be strictly interpreted as exploratory and hypothesis-generating rather than definitive. Our findings should therefore be validated in larger, multi-center cohorts. Second, due to the cross-sectional and descriptive nature of this research, we cannot establish causal relationships between the observed microbial profiles and the etiology of ASD or GI symptomatology.

Additionally, while the sibling-pair design controlled for many shared environmental factors, individual variables such as specific dietary patterns (e.g., food selectivity), lifestyle factors, and the use of pharmacological treatments (e.g., antipsychotics and melatonin) may have influenced the gut community structure. Given the pivotal role of diet in microbiome composition, we are currently working to incorporate detailed dietary intake data and anthropometric measures to enhance future comparative analyses.

In addition, we acknowledge that the high prevalence of polypharmacy in our ASD cohort (*n* = 11 out of 17 medicated participants) represents a significant confounding factor. While the co-occurrence of medications is visualized in **Figure 4A** our small sample size limits our ability to isolate the specific effects of individual drug classes or their combinations. Consequently, these findings should be interpreted as exploratory associations between the medicated state and the microbiota, rather than evidence of a causal relationship driven by a specific pharmacological agent.

## 6 Conclusion

This study provides a high-resolution characterization of the gut microbiota in Uruguayan children with ASD compared to their NT siblings. By utilizing a sibling-pair design, we effectively controlled for significant confounding variables such as environmental exposures, household conditions, and genetic background. In addition, the application of third-generation sequencing technology further allowed for improved taxonomic resolution, enabling a more precise identification of microbial signatures within a Latin American context.

Our findings reveal a significant depletion of key butyrate-producing taxa, specifically *Faecalibacterium* and *Coprococcus*, in children with ASD. Given the role of butyrate in maintaining the intestinal health, these deficiencies suggest a potential mechanism through which microbial dysbiosis may influence neurodevelopmental pathways. Additionally, the enrichment of the genus *Sellimonas* in the ASD cohort aligns with emerging evidence linking this taxon to gut-brain axis disorders.

The high prevalence of GI symptoms in our ASD cohort (93%) underscore the clinical relevance of the gut microbiome in managing these symptoms that often co-occur with autism. Furthermore, the observed reduction in the relative abundance of *Akkermansia* in children treated with antipsychotics and melatonin emphasizes that pharmacological interventions may account for the observed microbial variation and should be taken into consideration in clinical research.

Given the descriptive nature of this study, we cannot establish causal relationships. Nonetheless, our results suggest notable differences in the gut microbiome composition between both groups. These observations should be interpreted with caution, as several additional variables may also contribute to the patterns observed. Ultimately, our findings contribute to a deeper understanding of the biological heterogeneity within ASD and highlight potential targets for future personalized interventions and functional studies.

## Data Availability

The datasets presented in this study can be found in online repositories. The names of the repository/repositories and accession number(s) can be found in the article/**Supplementary Material**.
