# Supplementary material for: Gut microbiota and its association with gastrointestinal symptoms and pharmacological treatments in a sibling-matched cohort with autism spectrum disorder
Source: Front Microbiomes. 2026 Mar 24;5:1777385. doi: 10.3389/frmbi.2026.1777385 (PMC13055624; doi:10.3389/frmbi.2026.1777385)
Supplement: Supplementary file 1 [file Supplementaryfile1.docx]

***Supplementary Material***

# Supplementary Figures


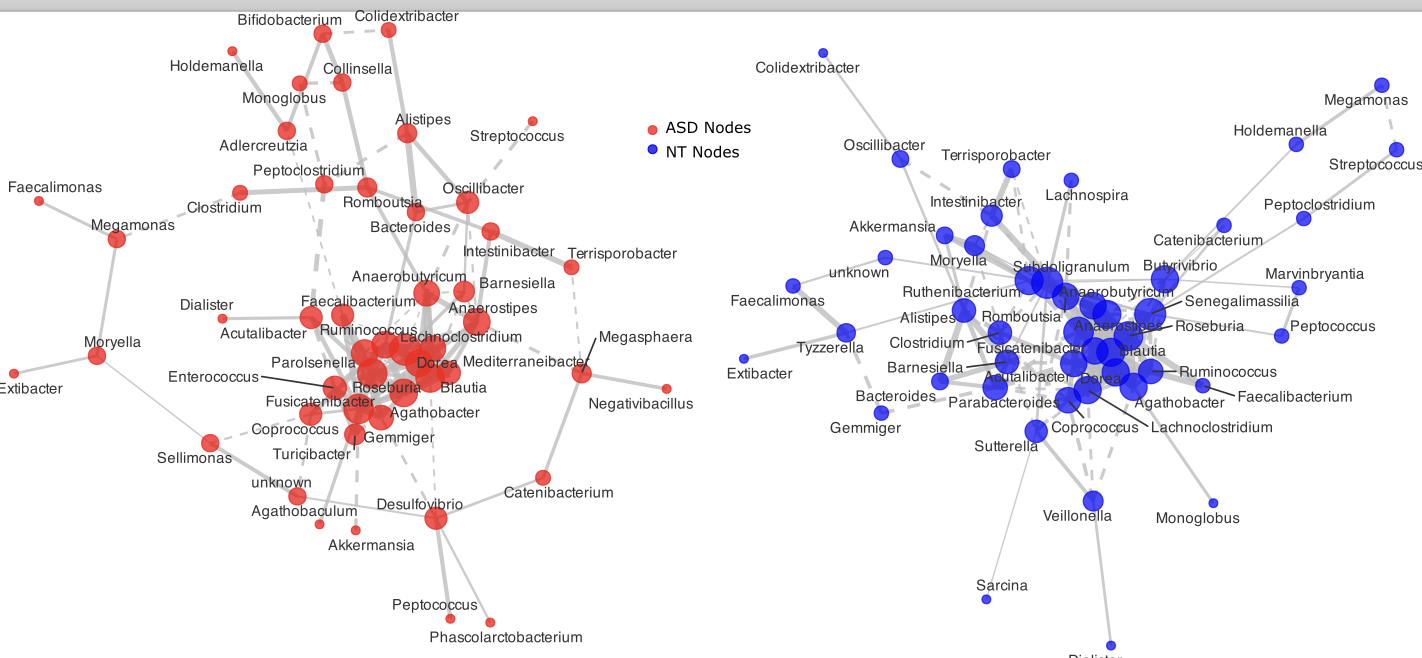


**Supplementary Figure 1**. Co-occurrence networks display bacterial interactions for ASD (red) and NT (blue) cohorts. Larger nodes indicate higher degree centrality (network hubs). Edges represent correlations; thickness corresponds to the correlation coefficient (*r*), while dashed and solid lines indicate negative and positive associations, respectively.


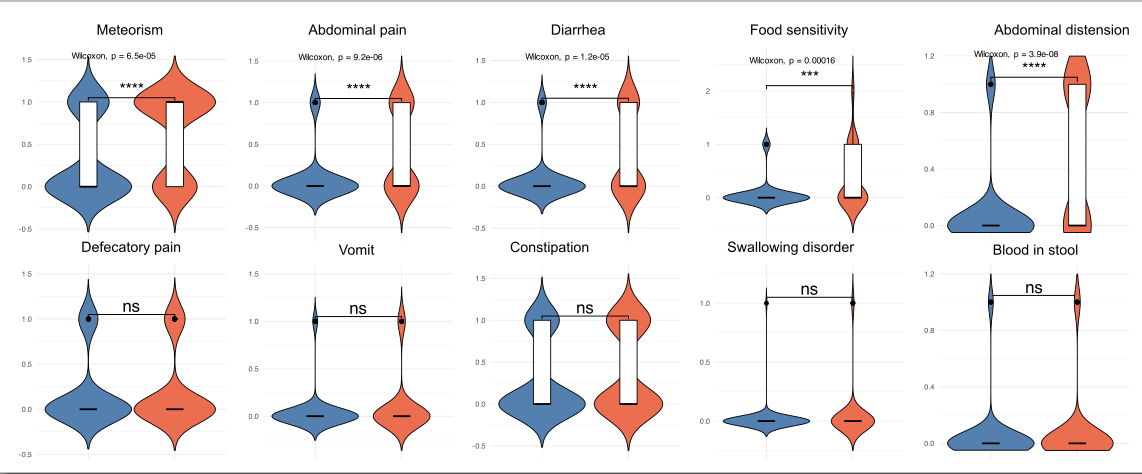


**Supplementary Figure 2.** Violin plots illustrate the distribution of symptom severity for ASD (red) and NT (blue) groups. Internal white boxes represent the interquartile range (IQR), with the horizontal black line denoting the median. Statistical significance was determined using the Wilcoxon rank-sum test; significant differences are marked with asterisks, while ns indicates non-significant results.


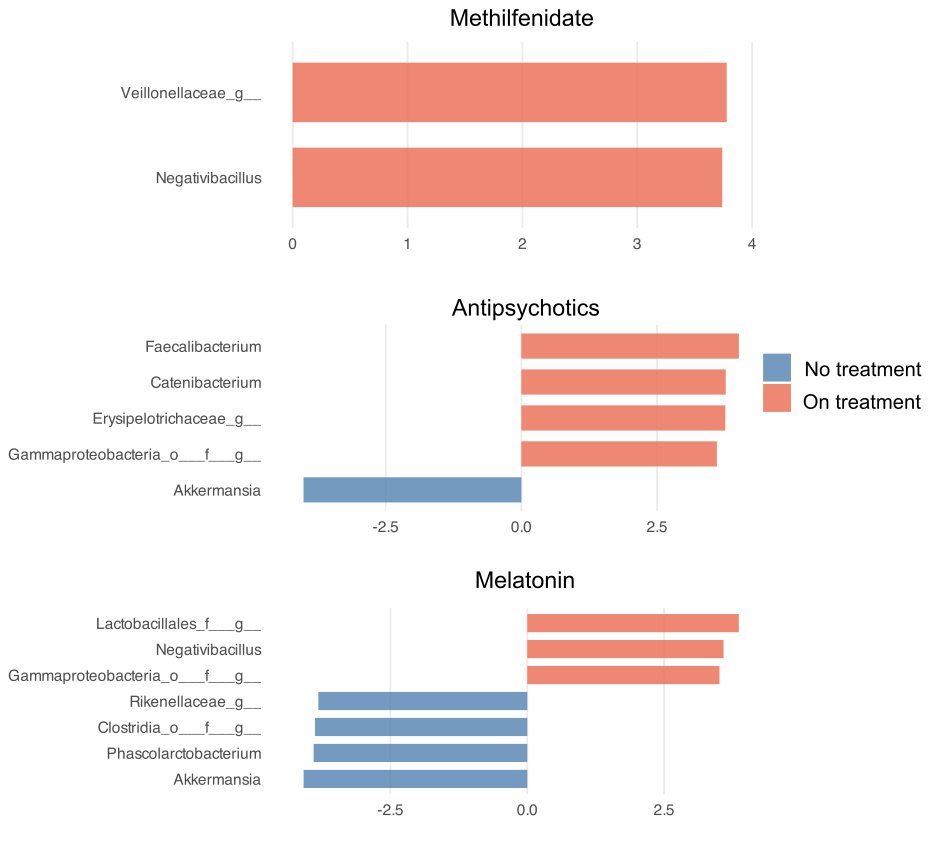


**Supplementary Figure 3.** Linear Discriminant Analysis Effect Size (LEfSe) results according to the pharmacological treatments. LDA scores show enriched taxa in each group, bars are colored according to this consumption.


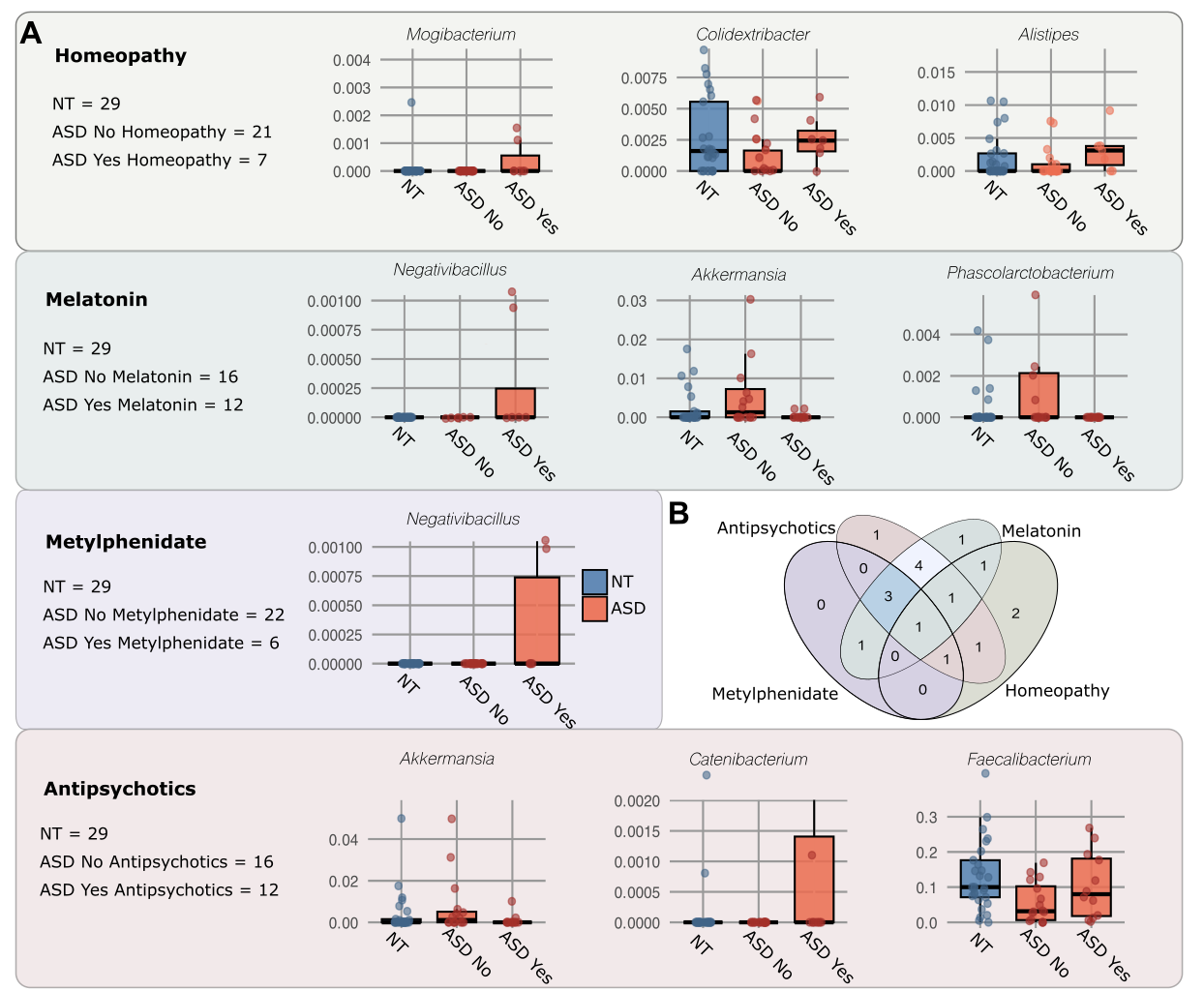


**Supplementary Figure 4.** Following a LEfSe analysis, the histograms show significant differential abundance of taxa associated with different consumption of Homeopathy.

# Supplementary Tables

**Supplementary Table 1.** **Lefse results with p values**

| FEATURE | feature | Enrich_  group | ef_lda | pvalue | padj | comparison |
| --- | --- | --- | --- | --- | --- | --- |
| marker1 | Faecalibacteriums | N | 3.918254 | 0.025108373 | 0.025108373 | NT vs ASD |
| marker2 | Coprococcuss | N | 3.620493 | 0.029055855 | 0.029055855 | NT vs ASD |
| marker3 | Sutterellaceae_g__s | N | 3.188932 | 0.040056436 | 0.040056436 | NT vs ASD |
| marker4 | Senegalimassilias | N | 3.184729 | 0.040056436 | 0.040056436 | NT vs ASD |
| marker5 | Lactobacillales_f___g__s | T | 3.689471 | 0.001779472 | 0.001779472 | NT vs ASD |
| marker6 | Sellimonass | T | 3.526663 | 0.02056364 | 0.02056364 | NT vs ASD |
| marker7 | Coriobacteriales_f___g__s | T | 3.487772 | 0.029414945 | 0.029414945 | NT vs ASD |
| marker8 | Bacilli_o___f___g__s | T | 3.425793 | 0.011075032 | 0.011075032 | NT vs ASD |

| Module | FEATURE | feature | Enrich_  group | ef_lda | pvalue | padj | comparison |
| --- | --- | --- | --- | --- | --- | --- | --- |
| Module 1 | marker1 | Anaerostipes | ASD_Absence | 4.309793 | 4.80E-02 | 4.80E-02 | ASD_Absence ASD_Presence  NT_Absence  NT_Presence |
| Module 1 | marker2 | Dialister | NT_Presence | 4.17E+00 | 4.05E-02 | 4.05E-02 | ASD_Absence ASD_Presence  NT_Absence  NT_Presence |
| Module 1 | marker3 | Coprococcus | NT_Presence | 4.03E+00 | 6.02E-03 | 6.02E-03 | ASD_Absence ASD_Presence  NT_Absence  NT_Presence |
| Module 1 | marker4 | Frisingicoccus | NT_Presence | 3.65E+00 | 6.23E-06 | 6.23E-06 | ASD_Absence ASD_Presence  NT_Absence  NT_Presence |
| Module 2 | marker1 | Oscillospiraceae_g__ | NT_Absence | 3.79905 | 0.028681425 | 0.028681425 | ASD_Absence ASD_Presence  NT_Absence  NT_Presence |
| Module 2 | marker2 | Clostridiales_f___g__ | NT_Absence | 3.572959 | 0.035222714 | 0.035222714 | ASD_Absence ASD_Presence  NT_Absence  NT_Presence |
| Module 2 | marker3 | Megamonas | NT_Presence | 3.807143 | 0.016346412 | 0.016346412 | ASD_Absence ASD_Presence  NT_Absence  NT_Presence |
| Module 2 | marker4 | Sutterella | NT_Presence | 3.418106 | 0.020689445 | 0.020689445 | ASD_Absence ASD_Presence  NT_Absence  NT_Presence |
| Module 2 | marker5 | Lactobacillales_f___g__ | TEA_Absence | 3.708848 | 0.007789938 | 0.007789938 | ASD_Absence ASD_Presence  NT_Absence  NT_Presence |
| Module 2 | marker6 | Bacilli_o___f___g__ | TEA_Absence | 3.582058 | 0.010639475 | 0.010639475 | ASD_Absence ASD_Presence  NT_Absence  NT_Presence |
| Module 3 | marker1 | Faecalibacterium | NT_Absence | 3.984256 | 0.0468956758 | 0.0468956758 | ASD_Absence ASD_Presence  NT_Absence  NT_Presence |
| Module 3 | marker2 | Acutalibacter | NT_Absence | 3.893037 | 0.0136511279 | 0.0136511279 | ASD_Absence ASD_Presence  NT_Absence  NT_Presence |
| Module 3 | marker3 | Coprococcus | NT_Absence | 3.799967 | 0.0422854 | 0.0422854 | ASD_Absence ASD_Presence  NT_Absence  NT_Presence |
| Module 3 | marker4 | Marvinbryantia | NT_Absence | 3.563572 | 0.0361903461 | 0.0361903461 | ASD_Absence ASD_Presence  NT_Absence  NT_Presence |
| Module 3 | marker5 | Sutterella | NT_Presence | 3.439839 | 0.0304282424 | 0.0304282424 | ASD_Absence ASD_Presence  NT_Absence  NT_Presence |
| Module 3 | marker6 | Lactobacillales_f___g__ | TEA_Absence | 3.675576 | 0.0163009042 | 0.0163009042 | ASD_Absence ASD_Presence  NT_Absence  NT_Presence |
| Module 3 | marker7 | Planococcaceae_g__ | TEA_Absence | 3.602184 | 0.0007364171 | 0.0007364171 | ASD_Absence ASD_Presence  NT_Absence  NT_Presence |
| Module 3 | marker8 | Psychrobacillus | TEA_Absence | 3.48987 | 0.0113014645 | 0.0113014645 | ASD_Absence ASD_Presence  NT_Absence  NT_Presence |
| Module 3 | marker9 | Epulopiscium | TEA_Absence | 3.464644 | 0.0113014645 | 0.0113014645 | ASD_Absence ASD_Presence  NT_Absence  NT_Presence |
| Module 3 | marker10 | Slackia | TEA_Absence | 3.45684 | 0.0343085698 | 0.0343085698 | ASD_Absence ASD_Presence  NT_Absence  NT_Presence |
| Module 3 | marker11 | Bacillaceae_g__ | TEA_Absence | 3.409958 | 0.0113014645 | 0.0113014645 | ASD_Absence ASD_Presence  NT_Absence  NT_Presence |
| Module 3 | marker12 | Bacillales_f___g__ | TEA_Absence | 3.281007 | 0.0007364171 | 0.0007364171 | ASD_Absence ASD_Presence  NT_Absence  NT_Presence |

**Supplementary Table 2. Pharmacological treatments and GI symptoms in the ASD group**

| **Medication Category** | **GI Symptoms (n = presence)** | **Participants (n)** | **Percentage (%)** |
| --- | --- | --- | --- |
| **No medication** | Constipation (n=6), Meteorism (n=5), Diarrhea (n=4), Abdominal pain (n=3), Distension (n=3), Food sensitivity (n=3), Vomiting (n=1), Blood in stool (n=1) | 14 | 48.3% |
| **Antipsychotics only** | Distension (n=2), Meteorism (n=2), Diarrhea (n=2), Abdominal pain (n=1), Food sensitivity (n=1), Swallowing disorder (n=1) | 2 | 6.9% |
| **Melatonin only** | Abdominal pain (n=1), Distension (n=1), Meteorism (n=1), Constipation (n=1), Food sensitivity (n=1), Blood in stool (n=1) | 2 | 6.9% |
| **Methylphenidate only** | None reported | 0 | 0% |
| **Antipsychotics + Melatonin** | Distension (n=4), Abdominal pain (n=3), Meteorism (n=5), Constipation (n=3), Food sensitivity (n=2), Blood in stool (n=2), Vomiting (n=1) | 5 | 17.2% |
| **Antipsychotics + Methylphenidate** | Meteorism (n=1), Diarrhea (n=1) | 1 | 3.4% |
| **Methylphenidate + Melatonin** | Distension (n=1), Meteorism (n=1) | 1 | 3.4% |
| **Triple Therapy (All three)** | Distension (n=3), Meteorism (n=2), Diarrhea (n=2), Abdominal pain (n=1), Constipation (n=1), Food sensitivity (n=1) | 4 | 13.8% |
| **Total** | - | **29** | **100%** |
